# The effects of handedness on sensorimotor rhythm desynchronization and motor-imagery BCI control

**DOI:** 10.1038/s41598-020-59222-w

**Published:** 2020-02-07

**Authors:** Dariusz Zapała, Emilia Zabielska-Mendyk, Paweł Augustynowicz, Andrzej Cudo, Marta Jaśkiewicz, Marta Szewczyk, Natalia Kopiś, Piotr Francuz

**Affiliations:** Department of Experimental Psychology, The John Paul II Catholic University of Lublin, Lublin, Poland

**Keywords:** Brain-machine interface, Human behaviour

## Abstract

Brain–computer interfaces (BCIs) allow control of various applications or external devices solely by brain activity, e.g., measured by electroencephalography during motor imagery. Many users are unable to modulate their brain activity sufficiently in order to control a BCI. Most of the studies have been focusing on improving the accuracy of BCI control through advances in signal processing and BCI protocol modification. However, some research suggests that motor skills and physiological factors may affect BCI performance as well. Previous studies have indicated that there is differential lateralization of hand movements’ neural representation in right- and left-handed individuals. However, the effects of handedness on sensorimotor rhythm (SMR) distribution and BCI control have not been investigated in detail yet. Our study aims to fill this gap, by comparing the SMR patterns during motor imagery and real-feedback BCI control in right- (*N* = 20) and left-handers (*N* = 20). The results of our study show that the lateralization of SMR during a motor imagery task differs according to handedness. Left-handers present lower accuracy during BCI performance (single session) and weaker SMR suppression in the alpha band (8–13 Hz) during mental simulation of left-hand movements. Consequently, to improve BCI control, the user’s training should take into account individual differences in hand dominance.

## Introduction

Brain-Computer Interfaces (BCI) are noninvasive systems that provide a channel of real-time communication and allow control of the external devices e.g. computers with no muscle activity. The input signal for the BCIs is the physiological data obtained by various neuroimaging methods. The data are transformed into output response of the effector. Almost 60% of BCI systems tested currently used electroencephalography (EEG) to register brain activity^1^. The most common EEG methods implemented for BCIs are based on sensorimotor rhythms (SMR) activity registered during imagery of movement^1^ and allow the design of so-called motor-imagery BCI (MI BCI) or sensorimotor rhythm-based BCI (SMR BCI). MI-BCI are experimentally used to control such devices as orthosis^2^, drones^3^, and wheelchairs^4^, as well as software for communication^5^.

Sensorimotor rhythms are brain oscillations registered during the preparation, execution, and imagery of a motor act at the electrodes placed over the sensorimotor cortex^6–10^. Two phenomena in SMR can be observed: a decrease of power during movement preparation or execution, which is the event-related desynchronization (ERD)^11^, and an increase of power after completing a movement, i.e., event-related synchronization (ERS)^12^. In the event of hand actual movement or imagery, there is a preponderance of contralateral ERD/ERS effect on central-parietal electrodes^7^. Duann and Chiou^13^ show that based on the independent components analysis (ICA) it is possible to identify sources related to ERD/ERS activity in the motor cortex. The lateralization of the ERD/ERS effect is used to control SMR-BCIs.

SMR can be separated into an alpha band (8–13 Hz) and a beta band (15–30 Hz), which display different functional properties within the sensorimotor system. Oscillations in alpha band enable functional coupling of remote cortical areas by the selection of task-relevant cortical regions, as well as for inhibition of activity in task-irrelevant regions^14^. Beta oscillations are engaged in control of muscular activity and communication between the cortex and periphery and related to some cognitive aspects of motor control, like visual cue anticipation and processing^15^. On the one hand, researchers state that the activity in beta bands simply reflects the maintenance of the current sensorimotor parameters and a cognitive states^16^ or activation/deactivation of motor cortical areas^17^. On the other hand, in the course of movement preparation, the ERD is regulated by uncertainty about the direction of an upcoming movement. The less confidence about the movement direction is associated with the reduction of ERD effect^18^.

The widespread usage of brain-computer interfaces encounters several obstacles. Among the most significant issues, two shall be highlighted, that is a low efficiency in translating brain activity into information and large individual differences in the ability to control effectively BCIs. Indeed, the reported phenomenon of BCI illiteracy^6^, indicates that a subject is unable to operate a given type of device at a rate higher than random. This problem is present in various BCI approaches in the group of 15–30% research participants. A lot of research currently focuses on inter- and intra-subject variation in BCI performance (see review by Ahn and Jun^19^). In recent years, the efforts to identify individual factors correlated with the BCI performance have yielded interesting results. The ability to control a SMR-BCI is moderated by motor experience, particularly the average number of hand-and-arm movements per day, practice in playing musical instruments^20,21^, frequency of manual activity^22^ and cognitive skills, e.g. visual-motor integration^23^ or mental rotation accuracy^24^. Vuckovic and Osuagwu^25^ reported that people with high BCI aptitude prefer kinesthetic rather than visual forms of motor imagery, measured by a self-reported questionnaire. However, this finding has not been confirmed in more recent studies^22^, so the estimation of the BCI performance using subjective methods could be ineffective.

In the study by Marchesotti *et al*.^26^, participants with a similar temporal profile, obtained in the mental chronometry task, showed both higher BCI abilities and stronger lateralization of sensorimotor rhythms during motor imagery. In fact, SMR lateralization is a major issue in the area of motor cognition research. Stancák and Pfurtscheller^27^ showed that hand dominance and handedness influence the lateralization of sensorimotor rhythms desynchronization during motor preparation. Right-handers exhibit stronger lateralization of SMRs preceding right-finger in comparison to left-finger movements, whereas in left-handed participants the similar contralateral preponderance for both sides was found. In another experiment, McFarland and colleagues^28^ reported differences between right- and left-hand movement or imagery in scalp topographies of SMR bands and individual differences in lateralization of the signal between the subjects. Still, there are no data indicating whether this pattern depends on the handedness of the subjects. Bai, Mari, Vorbach and Hallet^29^ investigated the patterns of ERD prior to sequential finger movements in the group of right-handers. They observed the contralateral prevalence of ERD only during right-hand finger movements, while ERD during left-hand finger movements was bilateral. Researchers also draw the conclusion that for the right-handers, the activation on the left hemisphere during non-dominant hand movements is greater than that on the right hemisphere during dominant hand movements. The neuroimaging studies also indicate that activation of motor areas during hand movements is different in right- and left-handed individuals, e.g. during sequential movement, the left-handers activate larger volumes and a larger number of brain areas than the right-handers. They also show significantly less brain lateralization; however, there are no such differences for simple movement^30^. In the other fMRI studies Pool *et al*.^31,32^ reported weaker asymmetry in the motor network effective connectivity in left-handers than right-handers during fist closures and a resting state.

To the best of our knowledge, there is no research directly investigating the effects of a subject’s handedness on SMR desynchronization in motor imagery tasks. On the one hand, the ERD/ERS pattern for left and right-hand imagery is well recognized and underlies SMR-BCI functioning, but the majority of research concerns only right-handed participants, as they represent a vast majority of the population^33,34^. To the extent that SMR-BCIs are based on recognizing the patterns of EEG activity during hand movement imagery, and the existing research proves dependence of SMR patterns in movement execution on handedness of the subjects, it seems crucial to investigate the role of handedness in SMR-BCI controls. For this purpose, we decided to use the ICA decomposition to identify the neural substrates of SMR activity evoked by the performance of the motor imagery task. Another essential value of our current study is that we investigate this issue based on an on-line BCI control task, while most of the studies use off-line data only, essentially relying on the classification of previously acquired signals without feedback. Moreover, we have decided to apply a complex and goal-oriented experimental procedure in order to eliminate random hits which could result in lower BCI accuracy. For this reason, the reported results may seem to be relatively low compared to typical offline or online studies (e.g. based on Graz BCI paradigm).

In this study, we hypothesized that handedness would influence both sensorimotor rhythm distribution during motor imagery and the accuracy of SMR-BCI control. We predicted that desynchronization of SMR would be more pronounced and BCI aptitude will be higher in right- than in left-handed individuals. Additionally, our goal was to determine whether there would be differences in ERD of SMR in motor imagery tasks, depending on an individual’s handedness and the estimated cortical location. We investigated the differences between the groups in a pure motor imagery task (off-line session) and a real-time BCI cursor control task (online session).

The description “Methods” can be found after the “Results” section.

## Results

### Off-line session: sensorimotor rhythms activity

**Figure 1:**
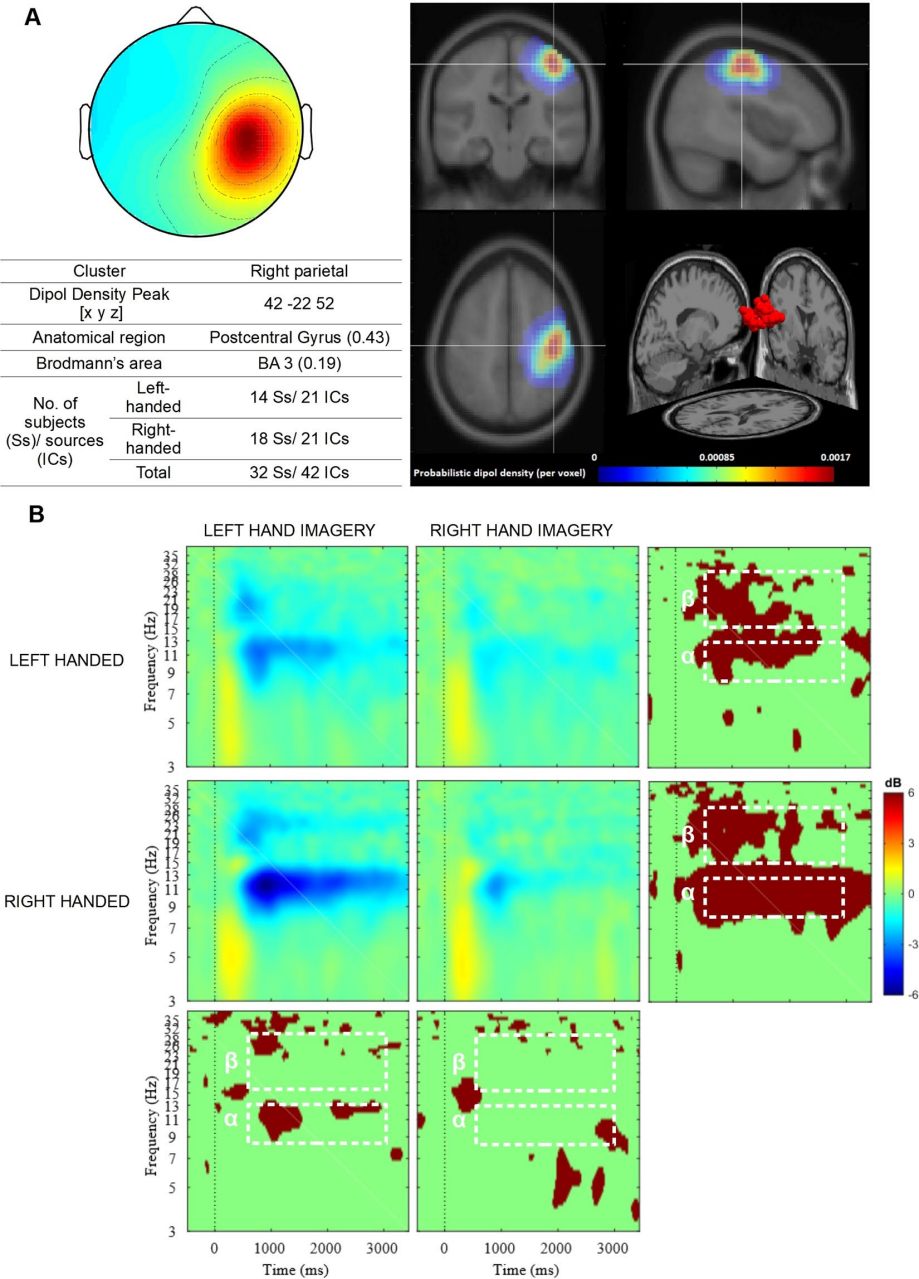
Right parietal cluster. (**A**) Average topography with the estimated anatomical location of the centroid (top left) and visualization of probabilistic dipoles density (top right); (**B**) Mean ERSP time x frequency spectrograms from all components for both experimental groups during left- and right imagery task. The plots of the bottom and right panel indicate the significant difference between conditions using a parametric test with a p-value of 0.05, no correction for multiple comparisons. White dotted lines indicate alpha (α) and beta (β) time-frequency regions that were averaged for the statistical analysis.

**Figure 2:**
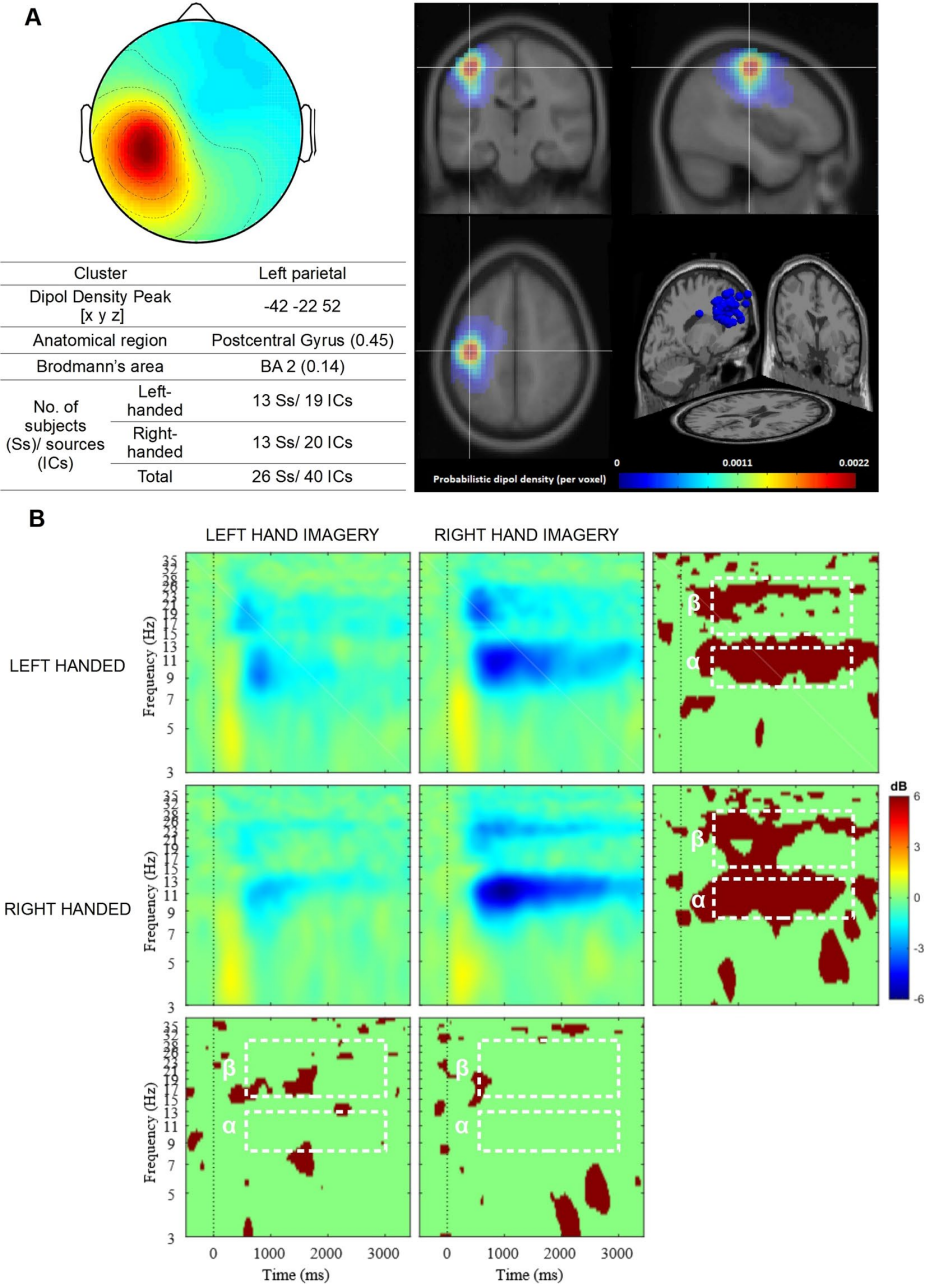
Left parietal cluster. (**A**) Average topography with the estimated anatomical location of the centroid (top left) and visualization of probabilistic dipoles density (top right); (**B**) Mean ERSP time x frequency spectrograms from all components for both experimental groups during left- and right imagery task. The plots of the bottom and right panel indicate the significant difference between conditions using a parametric test with a p-value of 0.05, no correction for multiple comparisons. White dotted lines indicate alpha (α) and beta (β) time-frequency regions that were averaged for the statistical analysis.

Fifteen clusters (548 ICs) were obtained after preprocessing of EEG data (Supplementary Fig. 1). The two of them (Cls 3 and Cls 7) were right and left parietal clusters with source estimated in right and left postcentral gyrus (Supplementary Fig. 2). These clusters showed stronger desynchronization of alpha and beta frequencies contralateral to the hand involved in the imagery task (Figs. 1B and 2B). Therefore, right and left parietal clusters were selected for analysis as representing SMR activities from the motor-related cortex.

### Right parietal cluster

A mixed design ANOVA was conducted with a between-subject factor HANDEDNESS (left-handed vs right-handed), within-subject factors IMAGERY TASK (left vs right-hand imagery) and FREQUENCY (8–13 Hz vs 15–30 Hz) and dependent variable SMR desynchronization (mean power decrease).

There were no main effects of the factors HANDEDNESS, *F*(1,40) = 1.47, *p* = 0.23, and interaction HANDEDNESS * FREQUENCY, *F*(1,40) = 0.01, *p* = 0.95. The main effect of the factor FREQUENCY was significant, *F*(1,40) = 13.11, *p* < 0.001, *η²* = 0.25, indicating that the mean power was lower in 8–13 Hz (*M* = −1.42 dB, *SE* = 0.31) than in 15–30 Hz (*M* = 0.88 dB, *SE* = 0.17). There was also a main effect of the factor IMAGERY TASK, *F*(1,40) = 22.66, *p* = 0.001, *η²* = 0.42. Desynchronization in alpha/beta bands is greater for left hand imagery than for right hand (left: *M* = −1.63 dB, *SE* = 0.26; right: *M* = −0.26 dB, *SE* = 0.26).

We found a significant interaction of FREQUENCY and IMAGERY TASK, *F*(1,40) = 10.07, *p* < 0.001, *η²* = 0.20. The Bonferroni post-hoc test (*p* < 0.001) showed greater desynchronization in 8–13 Hz (*M* = −2.03 dB, *SE* = 0.25) than in 15–30 Hz (*M* = −1.23 dB, *SE* = 0.15) during left hand imagery.

The interaction of the factors IMAGERY TASK and HANDEDNESS was significant, *F*(1,40) = 6.31, *p* = 0.016, *η²* = 0.14. The planned comparison test revealed that in the group of right-handed participants there was a significant difference in mean power decrease between imagery task conditions (*p* < 0.001). More specifically, there is a greater desynchronization during left hand imagery (*M* = −2.05 dB, SE = 0.26) than during right hand imagery (*M* = −0.64 dB, *SE* = 0.26) for the right-handers, while there is no such difference for the left-handers (*p* = 0.051). Also, the mean power decrease was greater for right-handed participants (*M* = −2.05 dB, SE = 0.26) than for the left-handers (*M* = −1.21 dB, *SE* = 0.26) during left hand imagery (*p* = 0.029).

**Figure 3:**
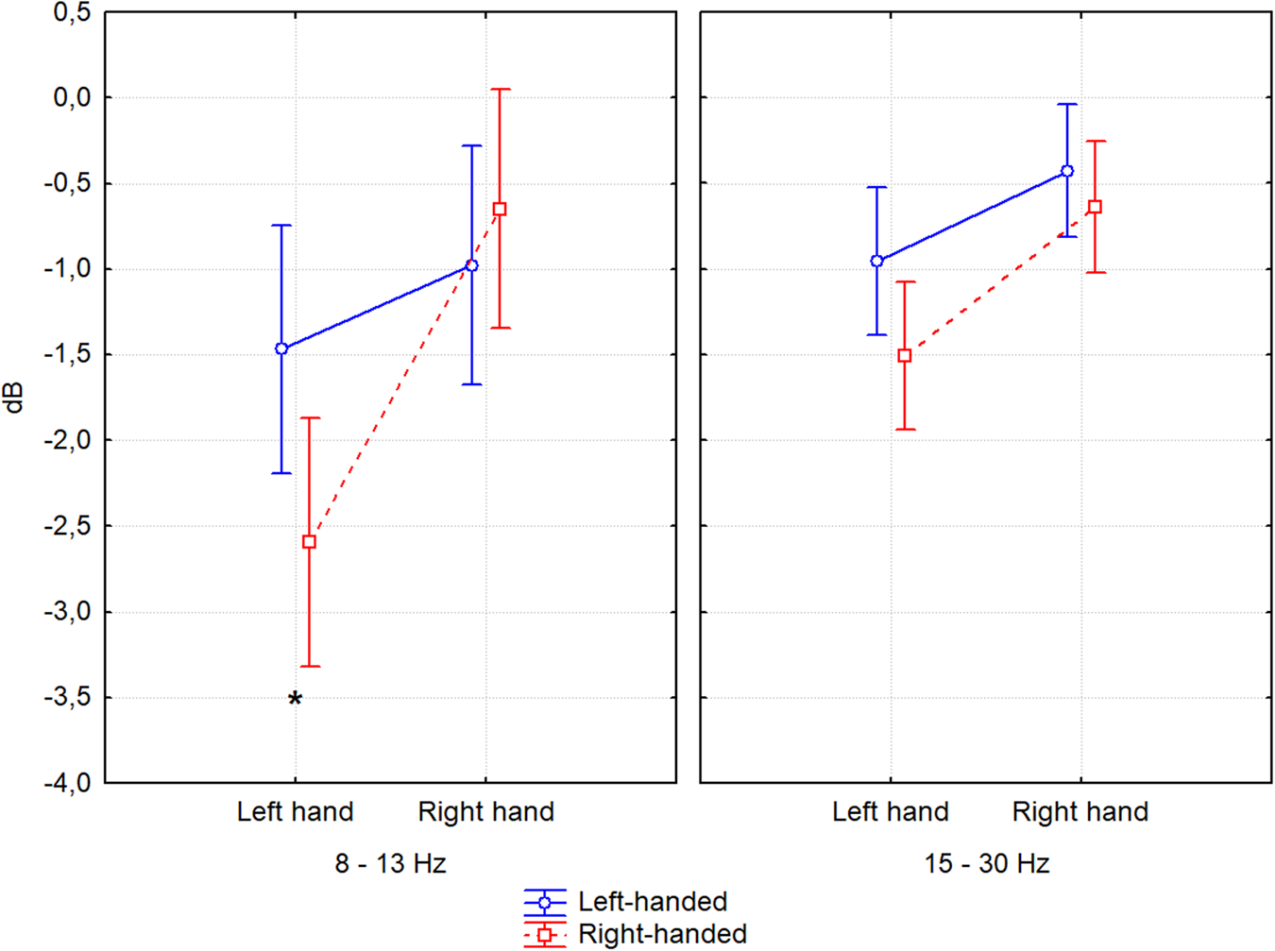
Differences in desynchronization of SMR (alpha and beta band) related to the effects of FREQUENCY × IMAGERY TASK × HANDEDNESS. The vertical bars represent standard error. Significant differences in planned comparison test are marked: *p < 0.05.

There was also another interaction with factor HANDEDNESS; we found that the FREQUENCY, IMAGERY TASK and HANDEDNESS interaction was significant, *F*(1,40) = 11.54, *p* = 0.001, *η²* = 0.22. The planned comparison test showed a difference in 8–13 Hz desynchronization between left and right-handers during left hand imagery *(p* = 0.031). Right-handed participants had greater alpha desynchronization (*M* = −2.59 dB, *SE* = 0.36) than left-handers (*M* = −1.46 dB, *SE* = 0.36) (Fig. 3).

### Left parietal cluster

A mixed design ANOVA was conducted with the between-subject factor HANDEDNESS (left-handed vs right-handed), within-subject factors IMAGERY TASK (left vs right-hand imagery) and FREQUENCY (8–13 Hz vs 15–30 Hz) and dependent variable SMR desynchronization (mean power decrease).

There were no significant main effects of HANDEDNESS, *F*(1,40) = 0.18, *p* = 0.64; and interaction: HANDEDNESS * FREQUENCY, *F*(1,40) = 0.22, *p* = 0.64. The two-way interaction of HANDEDNESS, FREQUENCY and IMAGERY TASK was also no significant *F*(1,40) = 0.78, *p* = 0.38.

We found a significant main effects of FREQUENCY, *F*(1,40) = 9.91, *p* = 0.003, *η²* = 0.20; IMAGERY TASK, *F*(1,40) = 30.91, *p* < 0.001, *η²* = 0.44 and interaction FREQUENCY * IMAGERY TASK, *F*(1,40) = 9.99, *p* = 0.003, *η²* = 0.20. Desynchronization was greater in 8–13 Hz (*M* = −1.56 dB, *SE* = 0.25) and when participants imagined a right hand movement (*M* = −1.79 dB, *SE* = 0.23) more than in 15–30 Hz (*M* = 0.99 dB, *SE* = 0.12) or during left hand imagery (*M* = −0.77 dB, *SE* = 0.16).

### On-line session: BCI performance

**Figure 4:**
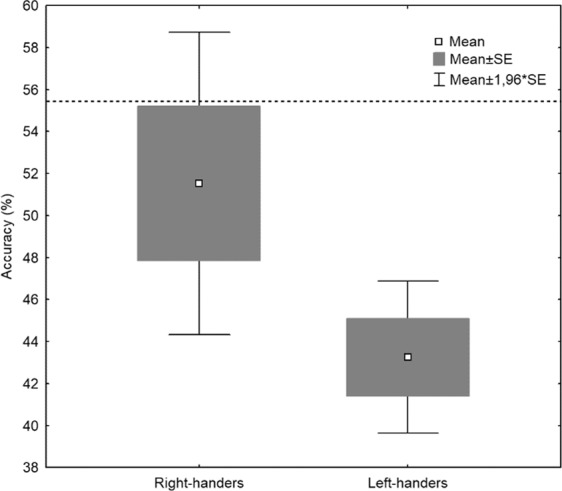
The accuracy (presented in %) in the BCI control task with regard to the HANDEDNESS. The dotted line indicate the upper confidence limits of chance calculate according to Müller-Putz and colleagues^61^ (results are shown for α = 5%).

An independent samples t-test, one-tailed was used to compare BCI performance in two experimental groups. The accuracy of control (% of correct hits) was subjected to a between-group analysis with the factor HANDEDNESS (left-handed vs right-handed). However, the assumption of homogeneity of variance was violated, Levene’s test: *F*(1.38) = 12.42; *p* = 0.001. In left-handed group, the assumption of normality was also violated, Shapiro-Wilk test: *W*(20) = 0.90; *p* = 0.036, while, in right-handed group, this assumption was retained, Shapiro-Wilk test: *W*(20) = 0.93; *p* = 0.173. Consequently, the one-tailed Welch’s t-test was used^35–37^. Additionally, Cohen’s d effect size was calculated and interpreted in accordance with Cohen’s^38^ guidelines: small *d* = 0.2, moderate *d* = 0.5, and large *d* = 0.8. There was a significant difference between left- and right-handed subjects in the percentage of correct hits, Welch’s t-test: *t* (28) = 2.02; *p* = 0.027. In the right-handed subjects there was a higher accuracy (*M* = 51.54%, *SD* = 16.43%) compared to the left-handed subjects (*M* = 43.25%, *SD* = 8.24%) (Fig. 4). Additionally, effect size was moderate, Cohen’s *d* = 0.64.

The accuracy of control from on-line session shows a negative correlation (Pearson’s *r* correlation, one-tailed) with alpha and beta frequencies from right and left parietal clusters: left hand imagery (Cls 3 *r*_*8–*1*3Hz*_ = −0.27, *p* = 0.042; *r*_15–30Hz_ = −0.32, *p* = 0.019) and right hand imagery (Cls 7 *r*_*8–13Hz*_ = −0.31, *p* = 0.025; *r*_*1*5–30Hz_ = −0.36, *p* = 0.011). This means that the participants’ better performance during BCI control coincided with a greater desynchronization of SMR recorded during offline session.

## Discussion

The results from off-line sessions show that right-handers had greater desynchronization of SMR from the right parietal cluster than left-handers during the left-hand motor imagery task. A significant difference was also observed between the SMR power for the right and left hand movement only in the right-handed group. In the left-handers, the suppression in right parietal cluster was similar for both hands. This effect can be attributed to a higher activation level of right sensorimotor cortex in right-handers. Also, Stancák and Pfurtscheller^27^ found differences between left- and right-handers in the mu rhythms domain. They explain the result basing on a neuroanatomical study^39^, the left-handers have a larger corpus callosum than the right-handers, which potentially facilitates bilateral hemispheric activation during motor acts. The fMRI studies confirm that brain activation and effective connectivity during sequential or skilled movements (praxis) and motor imagery is less asymmetric in left-handed individuals^30,32,40,41^. Moreover, during motor tasks left-handers exhibited activations in various areas that are not typically associated with motor control: V1, V2, auditory and prefrontal cortex^42^. This result, in particular, may point towards the different organization of the motor network in this group.

According to Marzoli *et al*.^43^, motor functions in left-handers are less lateralized compared to right-handers during the mental simulation of action. Another difference between these two groups is that left-handers rely more on a pictorial hand representation, whereas right-handers rely rather on a pragmatic hand representation^44^. Moreover, left-handed people seem to prefer more allocentric than egocentric perspective during e.g. mental rotation task^45^. In consequence, pictorial and allocentric representation of movement may involve more visual than sensorimotor processing and evoked different brain activity patterns.

According to Blankertz *et al*.^46^, Maeder *et al*.^47^, and the other authors (see review by Ahn and Jun^19^) SMR power from a Laplacian filtered channels (C4 and C3 areas) over the left and the right hemisphere is a good predictor of BCI classification and performance during a feedback session. Also, Marchesotti *et al*.^26^ showed that high-aptitude BCI users present higher suppression in the 8–12 Hz frequency range during an off-line motor imagery task. In our study, we observed similar effect, which is a correlation between accuracy of control in BCI session and alpha/beta desynchronization. Moreover, we found significant differences in overall MI BCI control accuracy between right- and left-handers. It supports our hypothesis that the right-handers could control BCI better than the left-handers. Most likely the right-handers involve more task-specific cortical activity of motor cortex in the BCI control task, which results in a higher success rate for guiding the ball left and right. The other factor that might contribute to this result arises from previous research, showing that people with high BCI aptitude prefer a kinesthetic rather than visual form of motor imagery and the right-handers rely on kinesthetic processes more than left-handers, while left-handers rely on visual processes more than right-handers^25,43^. Although we did not discriminate the kinesthetic and visual aspects of motor imagery, it would be valuable to include these properties of right- and left-handers in a future experiment in order to verify the potential interaction of these factors. At the same time, the kinesthetic rather than visual motor imagery (similar to the egocentric perspective) facilitates corticomotor excitability^48^ and produces a lateralized pattern of SMRs that can be classified by BCI algorithms^49^. The abovementioned neuroanatomical differences and distinct imagery strategies may explain why left-handers present weaker alpha suppression during simulation of left-hand movements and less accuracy during BCI performance.

The current study aimed at catching two aspects of the same issue, mainly the relation of sensorimotor rhythm desynchronization to handedness in a motor imagery task with feedback (BCI session) and without it (off-line session). Results indicate that the handedness of participants is of importance for both tasks. Additionally, we found the differences in mean power in 8–13 Hz frequency for the motor imagery task in right parietal cluster, but neither in left parietal cluster nor in beta band. This means that our results are limited to electrophysiological correlates of left-hand motor imagery in alpha/mu rhythms range. There are studies showing that in the beta band a similar, bilateral pattern can be observed at the motor areas during right and left-hand movement^50^, what is congruent with our result. In the alpha band, on the other hand, the effect indicating differences between the left- and right-handers in ability to produce symmetrical ERD patterns was observed at one side of the head only, while the effective MI BCI control requires distinction in signal between both sides. If we assume there is more involvement of task-specific cortical activity of motor cortex during the imagery task in the right-handers, we can hypothesize it resulted in better control over the ball in the MI BCI task. The significant differences in desynchronization between left and right motor imagery task in right-handers seem to be congruent with this result.

## Conclusions

The results of this study show that the distribution of sensorimotor rhythms during hand motor imagery is different for right- and left-handed subjects. We also demonstrated that handedness is closely linked to the ability to control an SMR-BCI. The unique value of this study stems from using an actual on-line BCI task, as opposed to a classified off-line signal, which further enhances the practicality and application of our results. These findings provide evidence against a hypothesis that BCI illiteracy can be completely ascribed to limitations of EEG in recording and processing brain signals. Our results imply that an assessment of hand dominance can be used as a predictor of future SMR-BCI performance and may thus help in designing more effective interfaces.

Some limitations of this study should also be mentioned. The off-line analyses concerned two ICs clusters located in the motor-related cortex and were reduced to the range of sensorimotor rhythms. It implies that the results and conclusions are also limited to this range of data. Another potential limitation is the fact that the motor imagery abilities can be distinguished between kinesthetic and visual aspects, which can also contribute both to the SMR patterns and BCI control results. Moreover, hand dominance is a complex issue and might be more continuous than a dichotomous phenomenon. Taking this into account, individual differences in motor imagery perspective (VMI and KMI) and diversity among right- and left-handed subjects should be controlled more precisely in future work.

More detailed studies on the relationship between types of hand preference and SMR activity during motor imagery would be interesting and of high value not only for the BCI research itself but also in the context of action representation and motor control issues.

## Methods

### Participants

40 BCI naive subjects (29 females) aged 18–41 yrs (*M* = 23.98; *SD* = 4.70) participated in the experiment. The left-handed group consisted of 20 subjects (14 females; aged 18–41 yrs; *M* = 25,10.67; *SD* = 5.99) and the right-handed group consisted of 20 subjects (15 females; aged 20–29 yrs; *M* = 22.85; *SD* = 2.62). All subjects performed the Edinburgh Handedness Inventory^51^, which assesses hand dominance. The more positive score of the inventory, the greater dominance of the right hand is, while the more negative score, the greater dominance of the left hand. To determine whether the groups demonstrate the dominance of left/right hand at a similar level, the Mann-Whitney *U* test was conducted. For scores below 0 (showing the dominance of left hand) the absolute values were used. In both the left-handed (*Me* = 90; *Q* = 13,75) and the right-handed (*Me* = 85; *Q* = 15) group the score of the inventory was high and the results did not differ between groups (*U* = 189.00; *p* = 0.758). Written informed consent was obtained from all participants that took part in the experiment. They also declared that they were neither taking medication nor any psychoactive substances on a permanent basis. At the end of the whole experimental procedure, participants were paid a remuneration of 60 PLN. The study was conducted in compliance with the Declaration of Helsinki and approved by the Ethics Committee of the Institute of Psychology at the John Paul II Catholic University of Lublin.

### Apparatus

The apparatus setting was taken from a similar study by Zapała and colleagues^52^ with two modifications: (1) in an off-line session 64-channel cap with active electrodes was used instead of 128-channel cap with passive electrodes, (2) in on-line session 8-cup active dry electrodes were used instead of 10-cup passive gel electrodes.

In an off-line session, changes in the activity of sensorimotor rhythms were measured with a GES 300 (Electrical Geodesics, Inc. Eugene, OR, USA) EEG system, comprising a Net Amps 300 amplifier (output resistance 200 MΩ; recording ranged from 0.01 to 1000 Hz) and a 64-channel actiCAP (Brain Products, Munich, Germany) cap with active electrodes. Electrode impedances were kept below 10 kΩ and the signal was referenced to an FCz channel during registration. Data sampling was defined at 500 Hz and recorded with a Net Station 4.4 (EGI, Eugene, OR, USA). The experimental procedure was designed and displayed on a screen with the use of E-Prime, version 2.0 (Psychology Software Tools, Pittsburgh, PA, USA).

The BCI on-line session was carried out using a Discovery 24E DC amplifier, from BrainMaster Technologies, Inc. (Bedford, OH, USA) with output resistance below 1000GΩ and an amplifier bandwidth of recording range of 0.000 Hz (DC) to 1000 Hz. We used 8-cup active dry electrodes (FC3, C3, C5, CP3, CP4, C4, C6, FC4) with a right-ear reference electrode and a ground electrode placed on a left ear from g.SAHARA systems (g.tec medical engineering GmbH, Graz, Austria). The sampling rate during recording was set to 128 Hz. Recording and processing of the data in online mode was carried out in OpenViBE 0.18.0 (Inria Hybrid Team, Rennes, France) During the off-line and online sessions, the stimuli were displayed on a LCD screen, with a diagonal measurement of 23 inches, and a resolution of 1920 × 1080 pixels. The subjects were comfortably seated at a distance of 60 cm from the monitor. Off-line signal processing of the EEG records was performed using EEGLab v12.0.2.6b, a toolbox to MATLAB 7.9.0 (MathWorks, Natick, MA, USA). Statistical analysis of the results was conducted with IBM SPSS 23PL.

### Procedure

The experiment comprised two stages: (1) the off-line session, which involved a recording of SMR patterns during a hand movement imagery task, (2) the online session – a BCI cursor control task with. Before the first session, the participants had received two rubber balls, one for each hand. They were instructed to perform the movement of repetitive clenching the balls and try to remember the accompanying physical sensations^49^. The online and off-line sessions were performed on different recording devices (see: Apparatus) and both of them were conducted on a single day. Between the sessions, there was a 30-minute planned break for washing and drying hair after the usage of gel electrodes and applying the dry ones. To avoid the interference of muscle activity in the EEG recording, the subjects were instructed to keep their hands relaxed and not to move it during the experiment. The position of the hands was observed by an experimenter from the control room during EEG registration.

### Off-line session: motor imagery task

**Figure 5:**
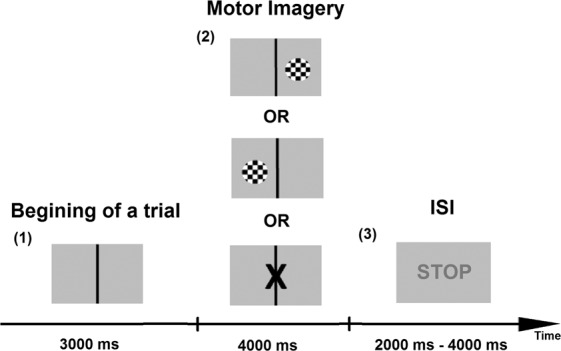
Off-line session procedure. (1) Baseline: the subject is not performing any activity; (2) Imagery: the display of the cue, which indicates what kind of movement should be imagined by the subject (left or right) or if subject should rest (3) The interstimulus interval (ISI): the end of the imagery task, ISI of the random length ranging between 2000–4000 ms.

The procedure for the EEG recording during a mental imagery task was adapted from a paradigm used by Hwang, Kwom and Im^53^. The data was recorded starting with a display of visual cues and continued until the end of the imagery task performance (Fig. 5). The participants were asked to imagine repetitive self-paced movements of their hand squeezing a ball, as they previously did during the preparation for the experiment. They were instructed to do it without any actual movement. During the off-line session, each subject performed a total of 180 trials (60 trials for each of the imagined left- and right-hand movements as well as the rest condition). The sequence of trials was randomized for each participant.

### Online session: BCI cursor control task

**Figure 6:**
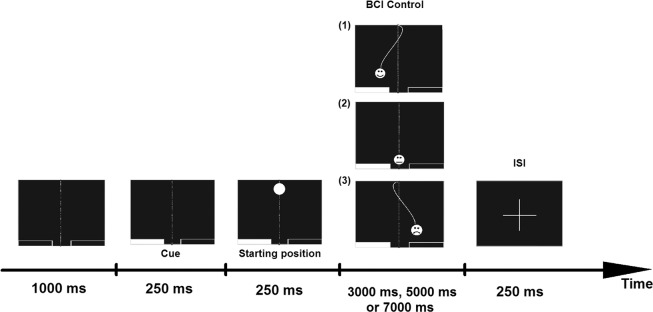
Online session procedure. The control stage presents possible user responses: (1) response matching the cue, (2) no response, (3) non-complying response.

The online session was a modified version of the procedure used by Krausz *et al*.^54^ and is shown in Fig. 6. The cursor control stage involved trials lasting for a few seconds (3, 5 or 7 seconds in random order), during which a participant was instructed to mentally perform left or right hand squeezing movements, similar to the off-line session. The aim of a task was to control a cursor (falling ball), directing it towards the basket on the screen. There were two baskets, one on the left side and the other on the right side of the screen. Each basket occupied 40% of the screen bottom with a space between them (the remaining 20% of the screen). The left-hand imagery directed the ball towards the left basket and the right-hand imagery towards the right one. The ball could either hit one of the baskets or miss, by simply falling down. Regardless of the result, the next trial started afterward. A short pause was followed by 40 trials (constituting a series). The length of the pause was controlled by the subject. During the session, a subject performed a total of 320 trials (160 for each hand) in 8 blocks.

### Data acquisition and analysis

#### Analysis of sensorimotor rhythms

The EEG signal in the off-line session was bandpass filtered in 1–40 Hz range using finite impulse response filter (FIR filter). A vertical and horizontal electrooculogram (EOG) was recorded to control for artifacts and a common average reference (CAR) was used on the other channels. Short-time high-amplitude artifacts have been removed by Artifact Subspace Reconstruction (ASR) method as implemented in Clean Raw Data plug-in^55,56^. Next, the ICA decomposition and equivalent current dipole model was conducted using *runnica* algorithm and boundary element head model (BEM), both implemented in the EEGLAB toolbox and DIPFIT plug-in^57^. The ICA components that may represent eye blinking, lateral eye movement, muscle activity, electrical noise, with a residual variance of dipole higher than 15% or locate outside of the brain were removed from the further analysis. The signal was divided into 6000 ms long segments (baseline from −2000 to −1000 ms, event period from 0 to 4000 ms) and subjected to time-frequency decomposition with Event-Related Spectral Perturbation^58^ using sinusoidal wavelet transformations (3-cycles; 0.5 s) in order to calculate the signal strength (dB) for entire window.

Remaining 548 ICs have been divided using *k*-means (*k* = 15) algorithm into 15 clusters based on feature vectors containing dipole locations, scalp projection maps and signal strength (8–30 Hz). Then the anatomical regions and Brodmann’s areas were estimated for each cluster based on dipole density (Supplementary Fig. 2). Based on mean time-frequency plots for all experimental conditions had been chosen the time window from 500 ms to 3000 ms in alpha (8–13 Hz) and beta (15–30 Hz) frequencies to averaging for statistical analyses.

In order to test the hypotheses, a two mixed design ANOVA was conducted for mean power strength (dB), representing SMR desynchronization in the event period (500–3000 ms) for the alpha (8–13 Hz) and beta (15–30 Hz) bands. The analysis was carried out separately for two ICs clusters: right (Fig. 1) and left (Fig. 2) parietal.

#### Analysis of BCI performance

Processing of the data in real-feedback mode was carried out according to the OpenViBE’s implementation of the Graz BCI paradigm “motor imagery with CSP filter”, described in detail by Suryotrisongko and Samopa^59^.

During the online session the signal was filtered with a bandpass filter for 8–30 Hz (Butterworth, order 5), divided into 1 s long epochs and the logarithmic band power was computed. The data were also subjected to a spatial filter, the Common Spatial Pattern (CSP), to improve efficiency in the discrimination of two classes of imaged movements. The signal classification was carried out with the use of Fisher’s Linear Discriminant Analysis (LDA)^60^ (Supplementary Fig. 3). The real-feedback procedure was based on 2D application coded in the C +  + programming language. Similar to the study by Krausz and colleagues^54^ the horizontal position of the ball was directly controlled by the LDA classification output signal to indicate the direction for displacement.

Behavioral data were acquired via automatic recording of the correct responses during BCI performance. Points were awarded if the ball hit any part of the highlighted area of the basket. No points were given if there was no response (the ball fell down into the area between the baskets) or if the ball was positioned in an area away from the highlighted basket^52^.

## Supplementary information

Supplementary materials.
