# Supplementary material for: The effects of handedness on sensorimotor rhythm desynchronization and motor-imagery BCI control
Source: Sci Rep. 2020 Feb 7;10:2087. doi: 10.1038/s41598-020-59222-w (PMC7005877; doi:10.1038/s41598-020-59222-w)
Supplement: Supplementary file 1 — Supplementary materials. [file 41598_2020_59222_MOESM1_ESM.pdf]

The effects of handedness on sensorimotor rhythm desynchronization and motor-imagery BCI control

Dariusz Zapała, Emilia Zabielska-Mendyk, Paweł Augustynowicz, Andrzej Cudo, Marta Jaśkiewicz, Marta Szewczyk, Natalia Kopiś, Piotr Francuz

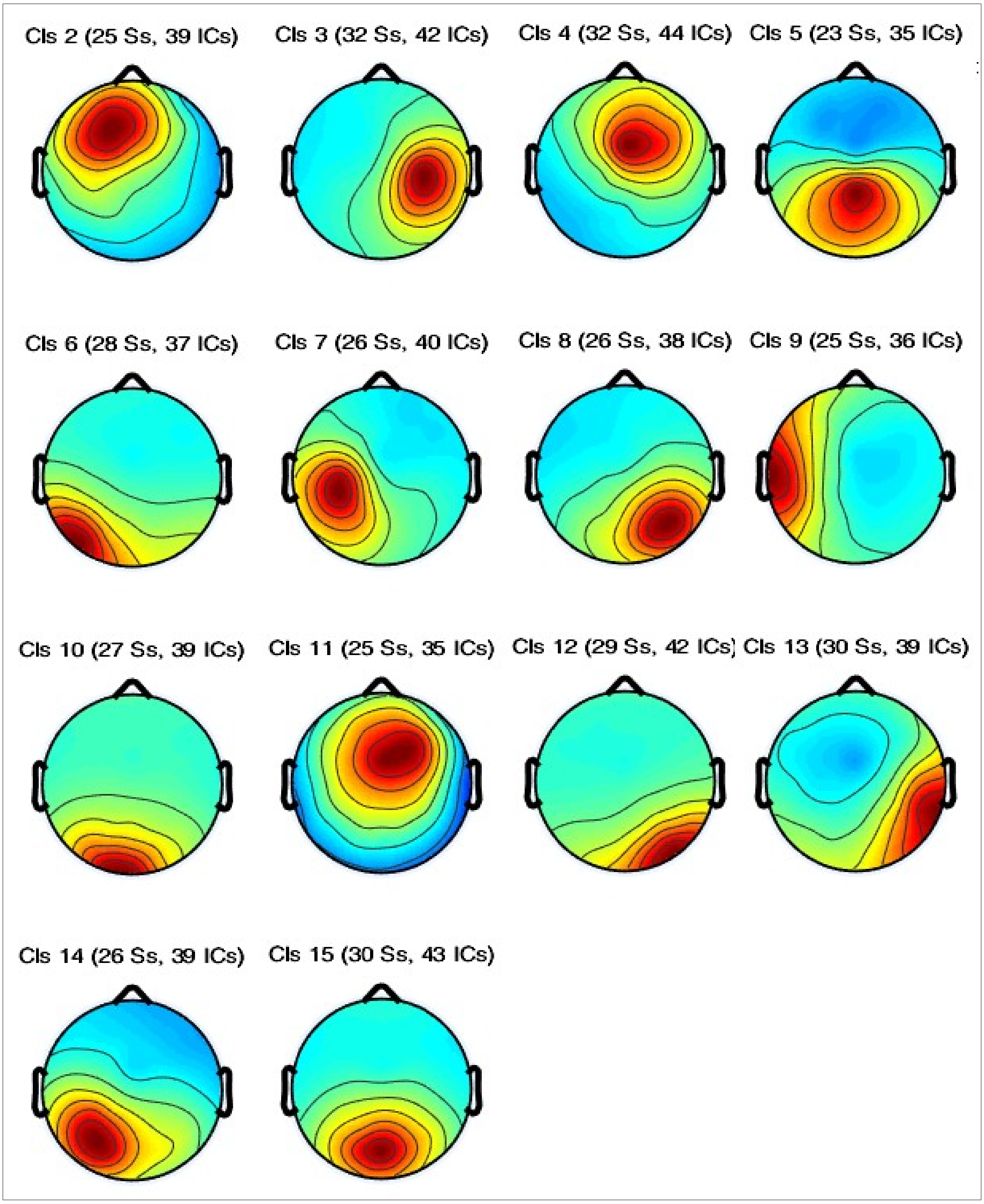

Supplementary Figure 1. Avarage scalp maps for all clusters. Ss – number of subjects; ICs – number of sources.

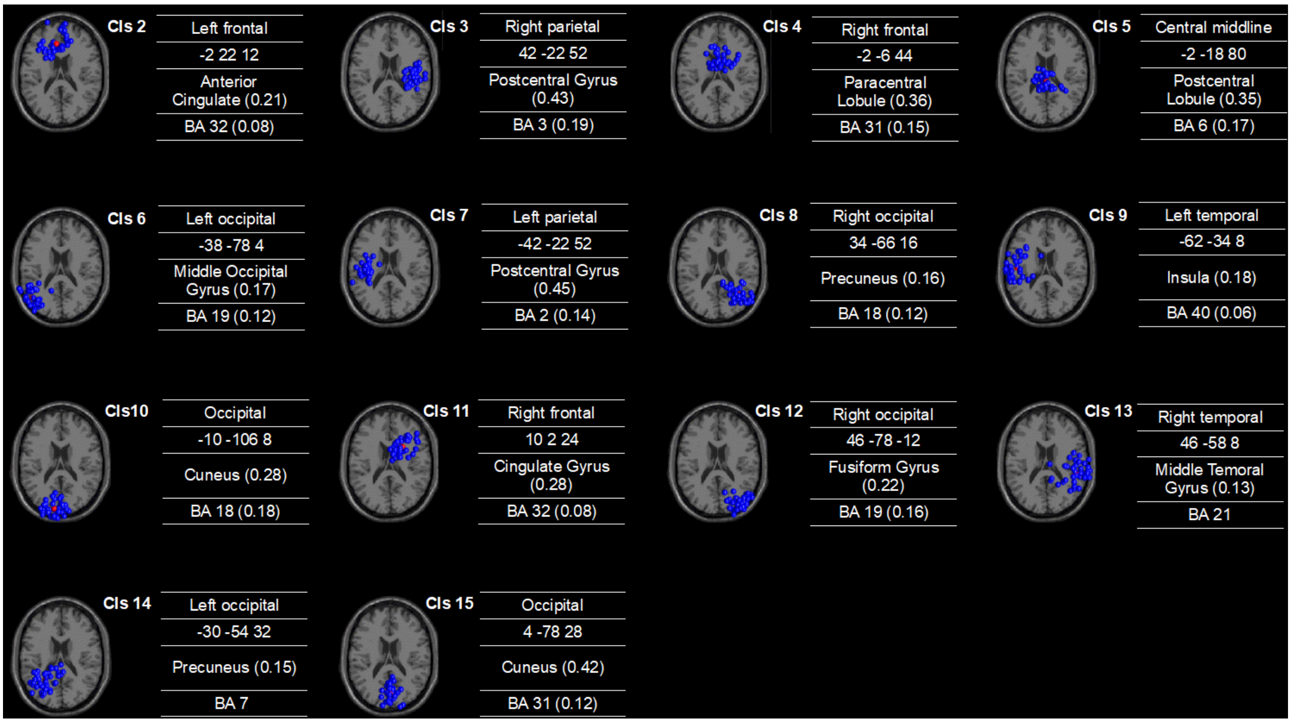

Supplementary Figure 2. Estimated dipole locations of ICs (*blue dots*) with MNI coordinates of cluster centroid and the probabilistic position in anatomical and Brodmann areas (*table*).

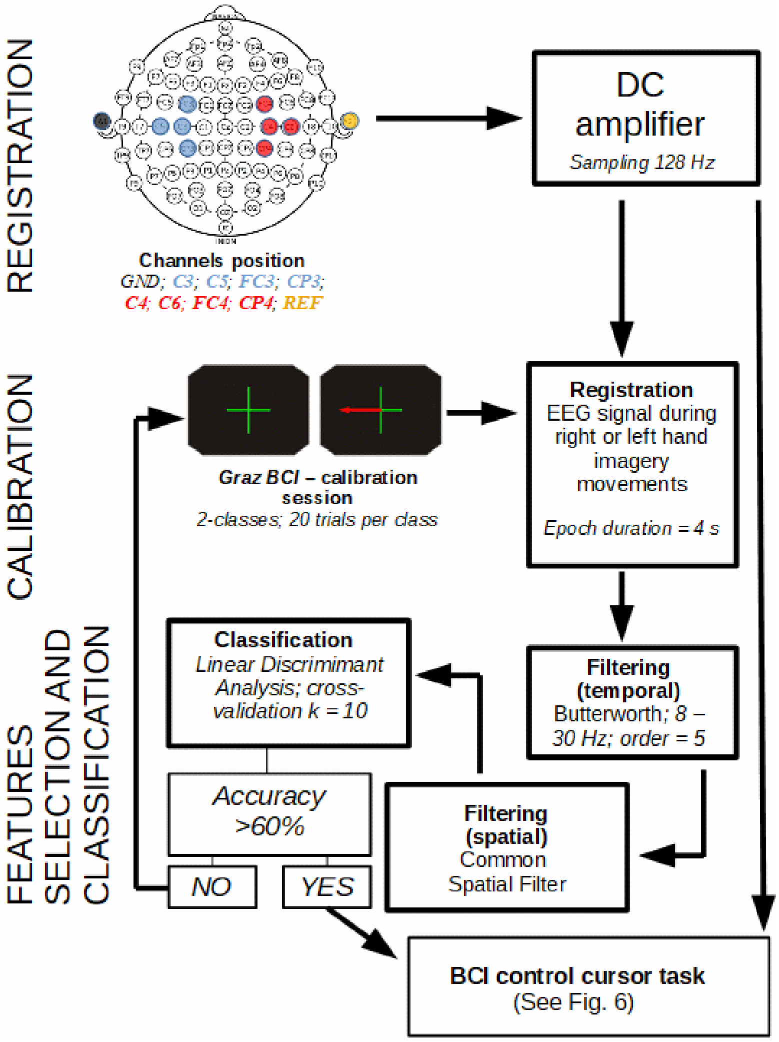

Supplementary Figure 3. The scheme of signal processing in the off-line session.
